# On the Ectrotic or Abortive Treatment of Gonorrhœa

**Published:** 1847-01

**Authors:** 


*On the Ectrotic or Abortive Treatment of Gonorrhœa.*—The employment of nitrate of silver in the early stages of gonorrhœa will, I also believe, prove highly serviceable, due regard being paid to the selection of cases in which trial of it is to be made. I have observed its operation on four occasions of distinct and undoubted clap, upon all of which the success attending its use was perfect. One of these was a first attack, open to objection on the ground of error in diagnosis, but so well marked in circumstantial evidence, as well as symptoms, as scarcely to be mistaken; two others were second attacks, thus less liable to fallacy; and the remaining one a fifth attack, in which, (to use the expressive language of the patient himself, "an old stager," in allusion to the existing ardor urinæ) "the red hot fish hooks were come." Nevertheless the complaint was as satisfactorily arrested in the last as in any of the previous cases, by the use of one injection only, although the patient admitted, that in no former attack, had the disease lasted upon him less than three months, notwithstanding the most assiduous attention to all the directions of a skilful medical man.

The mode of employing the remedy is simple. An injection composed of twelve grains of the salt to the ounce of water, is the proper strength to use. About a couple of drachms of this, by means of an ivory (or, for obvious chemical reasons, what is better, a glass) syringe, is to be thrown into the urethra, the penis being at the same time elevated and compressed at about two inches from the orifice, thus ensuring complete application of the solution to the urethral membrane within this range, and no further. The nozzle of the syringe being withdrawn, the orifice of the urethra is to be occluded, and the solution kept in contact with the mumous membrane for the space of not less than half a minute. No urine is to be passed for half an hour after the injection, and the penis is to be kept suspended. The immediate visible effect of the remedy is to form a coagulated film on the surface of the urethral lining; and this, undoubtedly, is a main agent in effecting the cure, by the protection it affords to the delicate and abnormally sensitive membrane during urinary evacuation. That it has this effect is evinced by the great diminution of pain which the patient at once experiences during micturition. But as to its *modus operandi*, we have also to consider its sedative action in subduing crescent inflammation, and its probable quality in neutralizing specific virus, and arresting zymotic increase.

The observance of rest and antiphlogistic regimen would, in all probability, aid the therapeutic influence of this mode of treatment; its beneficial effect is, however, developed under an ordinary mode of life, when attended with no flagrant violation of conduct.

As a method of cure, it is in my opinion open to but one objection, namely, its limited adoption, owing to its applicability extending no further than the early stages of the disease, and these often exciting but little attention. Let it not, however, be disregarded on this account; for, undeniably, many cases of clap present themselves while within the power of the remedy; and for these let it be reserved and had recourse to as a means easy of application and effectual in operation; while those cases beyond its influence may, as heretofore, be set aside, to be dealt with after another more expedient mode.

Failing, however, in ectrosis, the case is in a position no more unfavorable than if its cure had not been attempted; the ulterior effects and complications of the disease are likely to be in no degree more imminent or grave.—*London Lancet.*

